# Supplementary material for: Structural-functional brain network coupling during cognitive demand reveals intelligence-relevant communication strategies
Source: Commun Biol. 2025 Jun 4;8:855. doi: 10.1038/s42003-025-08231-4 (PMC12137705; doi:10.1038/s42003-025-08231-4)
Supplement: Supplementary file 2 — Reporting Summary [file 42003_2025_8231_MOESM2_ESM.pdf]

nature portfolio

Corresponding author(s): Johanna L. Popp, Kirsten Hilger

Last updated by author(s): May 12, 2025

Reporting Summary

Nature Portfolio wishes to improve the reproducibility of the work that we publish. This form provides structure for consistency and transparency in reporting. For further information on Nature Portfolio policies, see our Editorial Policies and the Editorial Policy Checklist.

Statistics

For all statistical analyses, confirm that the following items are present in the figure legend, table legend, main text, or Methods section.

n/a	Confirmed	
☐	☒	The exact sample size (*n*) for each experimental group/condition, given as a discrete number and unit of measurement
☐	☒	A statement on whether measurements were taken from distinct samples or whether the same sample was measured repeatedly
☐	☒	The statistical test(s) used AND whether they are one- or two-sided<br>*Only common tests should be described solely by name; describe more complex techniques in the Methods section.*
☐	☒	A description of all covariates tested
☐	☒	A description of any assumptions or corrections, such as tests of normality and adjustment for multiple comparisons
☐	☒	A full description of the statistical parameters including central tendency (e.g. means) or other basic estimates (e.g. regression coefficient) AND variation (e.g. standard deviation) or associated estimates of uncertainty (e.g. confidence intervals)
☐	☒	For null hypothesis testing, the test statistic (e.g. *F*, *t*, *r*) with confidence intervals, effect sizes, degrees of freedom and *P* value noted<br>*Give P values as exact values whenever suitable.*
☒	☐	For Bayesian analysis, information on the choice of priors and Markov chain Monte Carlo settings
☒	☐	For hierarchical and complex designs, identification of the appropriate level for tests and full reporting of outcomes
☐	☒	Estimates of effect sizes (e.g. Cohen's *d*, Pearson's *r*), indicating how they were calculated

Our web collection on statistics for biologists contains articles on many of the points above.

Software and code

Policy information about availability of computer code

Data collection	We used open access fMRI, DWI and behavioral data provided by the Human Connectome Project (HCP Young Adult 1200; Van Essen et al., 2013; https://doi.org/10.1016/j.neuroimage.2013.05.041) for our main analyses. The neuroimaging data was downloaded in the minimally preprocessed form (Glasser et al., 2013; https://doi.org/10.1016/j.neuroimage.2013.04.127). For the replication analyses, open access fMRI, DWI and behavioral data from the PIOP1 and PIOP2 samples provided by the Amsterdam Open MRI Collection (AOMIC; Snoek et al., 2021; https://doi.org/10.1038/s41597-021-00870-6) were used. Again, neuroimaging data were downloaded in the minimally preprocessed form.
Data analysis	Code for further preprocessing steps is available on GitHub: DWI preprocessing - https://github.com/civier/HCP-dMRI-connectome; fMRI preprocessing - https://github.com/faskowit/app-fmri-2-mat. Analysis code used in the current study is available on GitHub: Computation of latent g-factor - https://github.com/jonasAthiele/BrainReconfiguration_Intelligence; Operationalization of SC-FC coupling: https://github.com/brain-networks/local_scfc; Main analysis and replication analysis: https://github.com/johannaleapopp/SC_FC_Coupling_Task_Intelligence. The code specifically developed for the analysis presented in this paper has also been deposited on Zenodo (https://doi.org/10.5281/zenodo.15348080)

For manuscripts utilizing custom algorithms or software that are central to the research but not yet described in published literature, software must be made available to editors and reviewers. We strongly encourage code deposition in a community repository (e.g. GitHub). See the Nature Portfolio guidelines for submitting code & software for further information.

Data

Policy information about availability of data

All manuscripts must include a data availability statement. This statement should provide the following information, where applicable:

- Accession codes, unique identifiers, or web links for publicly available datasets
- A description of any restrictions on data availability
- For clinical datasets or third party data, please ensure that the statement adheres to our policy

Data from the main and the lockbox samples were obtained from the Human Connectome Project's 1200 Subjects Data Release (S1200) which can be accessed through the HCP data platform, ConnectomeDB, under https://www.humanconnectome.org/study/hcp-young-adult. Access requires registration and agreement to data use terms. Replication samples were drawn from the AOMIC and are publicly available through OpenNeuro at https://openneuro.org/datasets/ds002785/versions/2.0.0 (PIOP1) and https://openneuro.org/datasets/ds002790/versions/2.0.0 (PIOP2).

Research involving human participants, their data, or biological material

Policy information about studies with human participants or human data. See also policy information about sex, gender (identity/presentation), and sexual orientation and race, ethnicity and racism.

Reporting on sex and gender	Information on gender was provided as part of the open access behavioral data. Please refer to the original publications for further details (HCP: Van Essen et al., 2013; http://dx.doi.org/10.1016/j.neuroimage.2013.05.041; AOMIC: Snoek et al., 2021; https://doi.org/10.1038/s41597-021-00870-6).
Reporting on race, ethnicity, or other socially relevant groupings	No socially constructed or socially relevant categorization variables were used in this research. All analyses were controlled for the confounding variables age, gender, handedness and in-scanner head motion (operationalized as mean framewise displacement).
Population characteristics	All samples used in this study (HCP Young Adult sample; AOMIC PIOP1 and AOMIC PIOP2 sample) contain data from young healthy adults. In the AOMIC samples, only data from students of the Amsterdam University of Applied Sciences are included. Sample-specific characteristics: HCP: N = 764; 402 female; 697 right-handed; mean age = 28.6 years; age range = 22-36 years II AOMIC PIOP1: N = 126; 70 female; 112 right-handed; mean age = 22.2 years; age range = 18.25 - 26 years II AOMIC PIOP2: N = 180; 103 female; 160 right-handed; mean age = 21.91 years; age range = 18.25 - 25.5 years.
Recruitment	See above. For details please refer to the original publications from Van Essen et al. (2013; http://dx.doi.org/10.1016/j.neuroimage.2013.05.041) and Snoek et al. (2021; https://doi.org/10.1038/s41597-021-00870-6).
Ethics oversight	All ethical regulations relevant to human research participants were followed. Procedures of the HCP were authorized by the Washington University Institutional Review Board, while the ethical committee of the department of Psychology at the University of Amsterdam approved study protocols of the Amsterdam Open MRI Collection (PIOP1 EC number: 2015-EXT-4366, PIOP2 EC number: 2017-EXT-7568). All participants provided informed written consent in accordance with the declaration of Helsinki.

Note that full information on the approval of the study protocol must also be provided in the manuscript.

Field-specific reporting

Please select the one below that is the best fit for your research. If you are not sure, read the appropriate sections before making your selection.

☐ Life sciences ☒ Behavioural & social sciences ☐ Ecological, evolutionary & environmental sciences

For a reference copy of the document with all sections, see nature.com/documents/nr-reporting-summary-flat.pdf

Behavioural & social sciences study design

All studies must disclose on these points even when the disclosure is negative.

Study description	The study is qualitative and quantitative cross-sectional. We extracted structural brain network connectivity from diffusion weighted imaging and functional brain network connectivity from fMRI data acquired during resting state and cognitive tasks. We assessed the alignment of both modalities, the structural-functional brain network coupling (SC-FC coupling) with one similarity and three communication measures and related it to behavioral data (i.e., intelligence). We used open access DWI, fMRI and behavioral data provided by the Human Connectome Project (HCP Young Adult 1200; Van Essen et al., 2013; http://dx.doi.org/10.1016/j.neuroimage.2013.05.041) for our main sample. For replication analyses, we used open access DWI, fMRI and behavioral data from two samples (PIOP1 and PIOP2) provided by the Amsterdam Open MRI collection (AOMIC; Snoek et al., 2021; https://doi.org/10.1038/s41597-021-00870-6).
Research sample	All investigated datasets (HCP Young Adult Sample, AOMIC PIOP1 and AOMIC PIOP2 sample) contain data from healthy young adults. More specifically, the AOMIC samples only include data from students of the Amsterdam University of Applied Sciences of the University of Amsterdam. We used HCP data from 764 subjects (402 female; 697 right-handed; mean age = 28.6 years; age

range = 22-36 years). For replication, we used data from the AOMIC PIOP1 sample (N = 126; 70 female; 112 right-handed; mean age = 22.2 years; age range = 18.25 - 26 years) and the AOMIC PIOP2 sample (N = 180; 103 female; 160 right-handed; mean age = 21.91 years; age range = 18.25 - 25.5 years).

Sampling strategy	Since we only used open access data, details about the sampling strategy are provided elsewhere. Open access data from the Human Connectome Project was used for our main analysis. For details on imaging parameters please refer to Van Essen et al. (2013; http://dx.doi.org/10.1016/j.neuroimage.2013.05.041). For details on resting-state fMRI acquisition please refer to Smith et al. (2013; http://dx.doi.org/10.1016/j.neuroimage.2013.05.039), for details on task-induced fMRI and behavioral data acquisition, please refer to Barch et al. (2013; http://dx.doi.org/10.1016/j.neuroimage.2013.05.033). Replication analyses were conducted on open access data from the Amsterdam Open MRI collection. For details on fMRI and behavioral data acquisition procedures please refer to Snoek et al. (2021: https://doi.org/10.1038/s41597-021-00870-6).
Data collection	Details on data collection can be found in the references provided above.
Timing	Details on timing can be found in the references provided above.
Data exclusions	Participants with a) missing DWI, resting-state fMRI, or task fMRI, b) missing personality scores or missing cognitive measures from 12 tasks required to compute a latent intelligence factor, or c) a Mini-Mental State Examination score smaller than 27 (serious cognitive impairment) were excluded. According to Parkes et al. (2018; https://doi.org/10.1016/j.neuroimage.2017.12.073), participants with excessive in-scanner head motion measured by framewise displacement during any fMRI condition were also excluded: mean FD > 0.20 mm; proportion of motion spikes (FD > 0.25 mm) > 20 percent; any motion spikes > 5.00 mm. This resulted in a final sample of 764 participants in the main sample. In the replication samples, the same criteria (missing demographic, behavioral or neuroimaging data, excessive in-scanner head motion) were applied resulting in 126 participants from the AOMIC PIOP1 sample and 180 participants for the AOMIC PIOP2 sample.
Non-participation	n/a
Randomization	n/a

Reporting for specific materials, systems and methods

We require information from authors about some types of materials, experimental systems and methods used in many studies. Here, indicate whether each material, system or method listed is relevant to your study. If you are not sure if a list item applies to your research, read the appropriate section before selecting a response.

Materials & experimental systems

n/a	Involved in the study	
☒	☐	Antibodies
☒	☐	Eukaryotic cell lines
☒	☐	Palaeontology and archaeology
☒	☐	Animals and other organisms
☒	☐	Clinical data
☒	☐	Dual use research of concern
☒	☐	Plants

Methods

n/a	Involved in the study	
☒	☐	ChIP-seq
☒	☐	Flow cytometry
☐	☒	MRI-based neuroimaging

Plants

Seed stocks	n/a
Novel plant genotypes	n/a
Authentication	n/a

Magnetic resonance imaging

Experimental design

Design type	Diffusion weighted imaging; resting-state and task-induced fMRI
Design specifications	Open access neuroimaging data from the Human Connectome Project were investigated in our main analyses.

Design specifications	Specifically, we used data from diffusion weighted imaging, resting-state fMRI and task-induced fMRI (7 tasks): Working memory, gambling, motor, language processing, social cognition, relational processing, emotion processing.<br>Imaging parameters are again further described in Van Essen et al. (2013; http://dx.doi.org/10.1016/j.neuroimage.2013.05.041). Details on resting-state fMRI acquisition are reported in Smith et al. (2013; http://dx.doi.org/10.1016/j.neuroimage.2013.05.039), while details on task fMRI and behavioral data acquisition are referred to in Barch et al. (2013; http://dx.doi.org/10.1016/j.neuroimage.2013.05.033).<br>Data from the Amsterdam Open MRI collection was used for replication analyses.<br>Specifically, DWI data and fMRI data from resting state and task (PIOP1: Working memory, anticipation, emotion, face perception,gender-stroop; PIOP2: Working memory, emotion, stop-signal) were used.<br>FMRI and behavioral data acquisition procedures are reported in Snoek et al. (2021; https://doi.org/10.1038/s41597-021-00870-6).
Behavioral performance measures	In the main sample (HCP), a factor of general intelligence was derived from 12 cognitive measures via bi-factor analysis. In the replication samples from the AOMIC, intelligence was operationalized as sum score of the Ravens Progressive Matrices Test. Further details on acquisition of behavioral data can be found here: Barch et al. (2013; http://dx.doi.org/10.1016/j.neuroimage.2013.05.033) and Snoek et al. (2021; https://doi.org/10.1038/s41597-021-00870-6).

Acquisition

Imaging type(s)	Diffusion weighted imaging and functional MRI
Field strength	3T
Sequence & imaging parameters	HCP: FMRI scans were obtained using a gradient-echo EPI sequence in two sessions with multi-slice acceleration (TR = 720 ms; TE = 33.1 ms; 2-mm isotropic voxel resolution; flip angle = 52°; multiband acceleration factor = 8).<br><br>AOMIC: For the AOMIC PIOP1 sample, fMRI data from the resting-state scan and the face perception task were acquired with multi-slice acceleration (TR = 750 ms; TE = 28 ms; 3-mm isotropic voxel resolution; flip angle = 60°; multiband acceleration factor 3), while all other fMRI scans in in the AOMIC PIOP1 sample and the AOMIC PIOP2 sample applied sequential acquisition (TR = 2000 ms; TE = 28 ms; 3-mm isotropic voxel resolution; flip angle = 76.1°).
Area of acquisition	Whole-brain, only data of the cortex was used in our analyses

Diffusion MRI ☒ Used ☐ Not used

Parameters	HCP: We estimated structural connectivity based on minimally preprocessed DWI data (TR = 5520 ms; TE = 89.5 ms; 1.25 mm isotropic voxel resolution; multiband acceleration factor = 3; b = 1000, 2000, 3000 s/mm2; 90 directions/shell).<br><br>AOMIC: For both datasets, structural connectivity was computed based on one diffusion-weighted scan (median TR = 7387 ms; TE = 86 ms; 2-mm isotropic voxel-resolution; b = 1000 s/mm2; 32 directions/shell)

Preprocessing

Preprocessing software	HCP: The HCP DWI and fMRI data was downloaded in the minimally preprocessed form (Glasser et al, 2013: https://doi.org/10.1016/j.neuroimage.2013.04.127). Details on DWI preprocessing can be found on GitHub: https://github.com/civier/HCP-dMRI-connectome. In short, bias correction, modelling of white matter fibers via constrained spherical deconvolution (Tournier et al., 2007; https://doi.org/10.1016/j.neuroimage.2007.02.016) and tissue normalization (Dhollander et al., 2021) were performed according to the MRTrix pipeline (Tournier et al., 2019; https://doi.org/10.1016/j.neuroimage.2019.116137). The pipeline for further fMRI preprocessing is available on GitHub (https://github.com/faskowit/app-fmri-2-mat). Most importantly, according to Parkes et al. (2018; stretgy no. 6), 24 head motion parameters, eight mean signals from white matter and cerebrospinal fluid, and four global signals were regressed out and task-evoked neural activation was removed by simultaneously adding basis-set task regressors (Cole et al., 2019; https://doi.org/10.1016/j.neuroimage.2018.12.054).<br><br>AOMIC: AOMIC DWI and fMRI data were also downloaded in the minimally preprocessed form (fMRI data preprocessed using fMRIprep v1.4.1; Esteban et al., 2019; https://doi.org/10.1038/s41592-018-0235-4), and the same preprocessing steps as in the main sample were applied.
Normalization	HCP: Original EPI frames were resampled to atlas space including all transforms (motion, EPI distortion, EPI to T1w from FLIRT BBR, fine tuning of EPI to T1w with bbregister, nonlinear T1w to standard MNI 152). Further details are reported in Van Essen et al. (2013; http://dx.doi.org/10.1016/j.neuroimage.2013.05.041), Glasser et al. (2013; https://doi.org/10.1016/j.neuroimage.2013.04.127), or in the HCP manual: https://www.humanconnectome.org/storage/app/media/documentation/s1200/HCP_S1200_Release_Reference_Manual.pdf.<br><br>AOMIC: See above. Details can be found in the original publication (Snoek et al., 2021).
Normalization template	MNI 152
Noise and artifact removal	Preprocessing involved a nuisance regression strategy with 24 head motion parameters, eight mean signals from white matter and cerebrospinal fluid, and four global signals (Parkes et al., 2018; strategy no. 6; https://doi.org/10.1016/j.neuroimage.2017.12.073). For task-induced fMRI data, basis-set task regressors (Cole et al., 2019; https://doi.org/10.1016/j.neuroimage.2018.12.054) were used simultaneously with the nuisance regressors to remove mean task-evoked activations.

Volume censoring

We excluded complete data from subjects with high in-scanner motion (as operationalized with framewise displacement; FD) for any one of the conducted scans: mean FD > .2 mm, proportion of spikes (FD > .25 mm) > 20%, or any spikes above 5 mm.

Statistical modeling & inference

Model type and settings

Relationship between SC-FC coupling and intelligence on brain-average level: Partial Spearman correlations between measure-specific brain-average SC-FC coupling (operationalized as mean value across all region-specific coupling values that were determined by correlating regional connectivity profiles, i.e., matrix columns, of structural and functional connectivity matrices) with general intelligence scores. Correlations were controlled for influences of age, sex, handedness and in-scanner head motion (mean framewise displacement).

Relationship between SC-FC coupling and intelligence on a region-specific level: Two predictive modeling approaches (the Basic NMA Model and the Expanded NMA Model) were applied to predict individual intelligence scores from region-specific SC-FC coupling. More specifically, we developed 5-fold internally cross-validated prediction frameworks based on multiple linear regression with input features created by assigning coupling measures to brain regions based on their association with general intelligence scores. For further details, please refer to the Methods section of the paper or to Popp et al. (2024; https://doi.org/10.1016/j.neuroimage.2024.120563). We again controlled for influences of age, sex, gender, handedness and in-scanner head motion (mean framewise displacement).

Effect(s) tested

Relationships on the brain-average level were assessed for each coupling measure and each condition separately (yielding 4*8 partial correlations). For the second part of our analyses, one Basic NMA Model was constructed for each condition (8 in total) and one Expanded NMA Model, combining SC-FC coupling information across tasks, was constructed for each investigated sample (Main sample, lockbox sample, AOMIC PIOP1 sample and AOMIC PIOP2 sample).

Specify type of analysis: ☒ Whole brain ☐ ROI-based ☐ Both

Statistic type for inference

(See Eklund et al. 2016)

Not applicable as we did not perform voxel-wise or cluster-wise analyses.

Correction

To correct for multiple comparisons in the analysis on the brain-average level, we used the Bonferroni correction (four comparisons; significant p < .0125). The significance of the prediction models was assessed via permutation testing.

Models & analysis

n/a	Involved in the study
☐	☒ Functional and/or effective connectivity
☒	☐ Graph analysis
☐	☒ Multivariate modeling or predictive analysis

Functional and/or effective connectivity

Functional connectivity was operationalized as Fisher-z transformed Pearson correlations between regional activity timecourses. Structural connectivity matrices entailed SIFT2 streamline density weights between all possible pairs of brain regions. SC-FC coupling was operationalized by transforming individual structural connectivity matrices into one similarity and three communication matrices, while consequently computing the Pearson correlation between regional connectivity profiles (matrix columns) of the similarity/communication matrix and the condition-specific functional connectivity matrix. These region-specific SC-FC coupling values, computed for each coupling measure (i.e., coming from the similarity/communication measures) and condition, were used in further analyses.

Multivariate modeling and predictive analysis

We predicted individual intelligence scores from region-specific SC-FC coupling values using a recently developed prediction framework (Popp et al., 2024; https://doi.org/10.1016/j.neuroimage.2024.120563).

The Basic Node-Measure Assignment Model (B-NMA) is based on multiple linear regression and explicitely considers region-speific variations in SC-FC coupling. It was run once for each condition and cross-validated via 5-fold internal cross-validation. In brief, for each coupling measure separately, region-specific coupling values from participants of a training sample were correlated with general intelligence scores (partial correlations controlling for effects of age, gender, handedness, and in-scanner head motion). This resulted in four correlation coefficients per brain region. Two group-general node-measure assignment (NMA) masks were then constructed by assigning the coupling measure with the largest positive magnitude association (positive NMA) and the coupling measure with the largest negative magnitude association (negative NMA) between intelligence and coupling strength to a given region. These masks were finally used to extract individual region-specific coupling values and two model input features were computed, one as average across all individual's coupling values extracted with the positive NMA and one as average across all individual's coupling values extracted with the negative NMA. General intelligence scores were then predicted with this model for each participant by using a 5-fold cross-validation scheme.

The Expanded NMA Model (E-NMA) is highly similar with respect to this methodology but combines region-specific SC-FC coupling information across all tasks: two model input features were created for each fMRI condition (excluding resting state) in a similar fashion, but these 14 condition-specific features were now considered simultaneously within the regression model to predict intelligence.

Model performance was determined as Pearson correlation between predicted and observed intelligence scores. For robustness, prediction performance was averaged across 100 repetitions with different training-test splits. Statistical significance was examined with non-parametric permutation tests (1000 iterations; $p < .05$).

To assess the generalizability of our model, the 5-fold internal cross-validation was also performed in the lockbox sample and the two independent samples from the AOMIC. Further, cross-sample model generalization tests were conducted: Main sample data were split into five folds, and models were trained on 80% of the data (4/5 folds). This resulted in five prediction models for each of the 100 training-test data splits, which were then used to predict general intelligence in the lockbox and replication samples. The performance of each cross-sample model generalization test was assessed by averaging across all prediction performance outcomes and significance was assessed via permutation testing.
